# Investigation into the use of a MOSFET dosimeter as an implantable fiducial marker

**DOI:** 10.1120/jacmp.v10i1.2893

**Published:** 2009-01-27

**Authors:** Stephen F Kry, Michael Price, Zhonglu Wang, Firas Mourtada, Mohammad Salehpour

**Affiliations:** ^1^ Department of Radiation Physics The University of Texas M. D. Anderson Cancer Center Houston TX U.S.A.

**Keywords:** fiducial marker, implantable dosimeter, *in vivo*, organ motion, image‐guided radiotherapy

## Abstract

It may be possible to use a single device to measure the *in vivo* dose delivered during radiotherapy, as well as to localize the target volume. This potential, as well as the detectors' ability to relate dosimetry and localization, were evaluated using two implantable MOSFET dosimeters placed inside an acrylic pelvic phantom. A wedged‐field photon plan and an eight‐field prostate treatment plan were developed. For each plan, conditions were simulated so that detectors were in their correct positions or slightly displaced to represent patient setup error and/or organ motion. Doses measured by the two detectors after irradiation were compared to those calculated by the treatment planning software. Additionally, using localization software and kilovoltage images of each setup, the displacement of the detectors from their correct locations was calculated and compared to the induced physical displacement. For all alignments and detector positions, measured and calculated doses showed an average disagreement of 2.7%. The detectors were easily visualized radiographically and the induced detector displacements were typically recognized by the localization software to within 0.1 cm. The implantable detector functioned well as both an internal dosimeter and as an internal fiducial marker, and thus may be useful as a clinical tool to localize the target volume and verify dose delivery *in vivo*.

PACS numbers: 87.53.‐j 87.55.‐x

## I. INTRODUCTION

The *in vivo* dose delivered during radiation therapy is a critical concern. However, because of the difficulties associated with making *in vivo* measurements, they are not frequently done. Furthermore, *in vivo* measurements that have been done^(^ ^1^ ^–^ ^4^ ^)^ typically suffer from limitations. First, they are usually done for only a limited number of treatment fractions, rather than for all the fractions of the treatment plan. Second, the measurement is usually only a surrogate for the dose to the target volume or structure of interest because the dose is usually measured on the patient's surface or within an air cavity, rather than directly at the target. A technique for measuring the *in vivo* dose was recently developed that overcomes both of these limitations: an implantable dose verification system (DVS) detector (Sicel Technologies, Morrisville, NC), which uses a metal oxide semiconductor field effect transistor (MOSFET). The dosimeter is surgically implanted directly into the target (or neighboring tissues of interest) and reports the dose delivered directly to the target after every treatment fraction. The dosimeter has shown reasonable accuracy and precision and has been used in preliminary clinical studies.^(^ ^5^ ^–^ ^9^ ^)^

Another critical issue in radiation therapy is target localization. Substantial interfractional target motion has been repeatedly observed^(10)^ ^,^ ^(11)^ and a variety of techniques are currently employed to correct for it, including the use of implanted fiducial markers^(12)^ ^,^ ^(14)^ to help track the target. The implantable DVS dosimeter may, in fact, be able to serve this second function as well. In addition to measuring the dose delivered to the target, it may also function as a fiducial marker because it has radio‐opaque components. However, the DVS dosimeter's potential role as a fiducial marker requires verification because the radio‐opaque structures in the dosimeter, including electronic chips and an antenna, have complex shapes and therefore may not be reproducibly resolved.

These two clinical objectives – verifying dose delivered to the target and localizing the target during treatment – are actually the same not determining the actual location of the target but rather evaluating and/or avoiding the dosimetric errors caused by mislocalization. Therefore it would be expedient to have a single integrated implant serve both roles. However, to the authors' knowledge, this has not been tried. The use of the implantable DVS detector as both a dosimeter and a fiducial marker could greatly increase the knowledge of a treatment as it is actually delivered to the patient. By imaging and verifying the location of the dosimeter (and, therefore, the target) before each irradiation, it may be possible to not only verify the dose delivered to the patient when the patient is properly aligned but also determine *in vivo* the dosimetric impact of any misalignments, residual setup errors, or geometric distortions of the target.

The current study was an evaluation of the dosimetric and fiducial marker capabilities of the implantable DVS detector. Furthermore, the utility of this dual dosimeter/localizer capability was investigated by evaluating the interrelatedness of the dosimetry and localization results in phantom studies where misalignments and internal motion were introduced in the phantom.

## II. MATERIALS AND METHODS

### A. Detectors and Phantom

**Figure 1:**
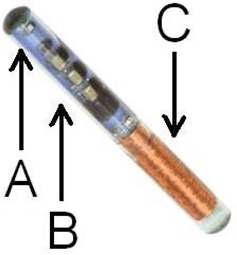
Implantable DVS detector (photo courtesy of Sicel Technologies), including (A) MOSFETs, (B) electronic chips, (C) antenna.

In this study, we used the most recent generation of DVS detectors (which are smaller than the previous version) measuring 20 mm long by 2.1 mm in diameter, and which are commercially available. The new design incorporates two MOSFETs in each DVS detector to minimize isotropy effects^(6)^ and uncertainties in the readings (Fig. 1). The two MOSFETs, each with an active area of 690×15μm,^(5)^ are placed back‐to‐back to minimize the angular dependency. The dosimeters are precalibrated for use at body temperature. Each dosimeter includes a calibration certificate and expected performance that can vary with lot production. The manufacturer specified dosimetric accuracy of the new DVS is <5.5% (2σ) up to a total dose of 20 Gy, and <6.5% (2σ) up to a total dose of 74 Gy.

**Figure 2:**
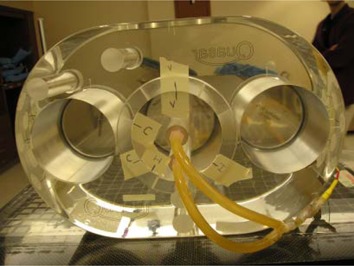
Acrylic pelvic phantom with a center circular insert that can be rotated. Rubber tubing circulates heated water over the center detector (in the center of the insert) and the edge detector (at the bottom edge of the insert).

**Figure 3:**
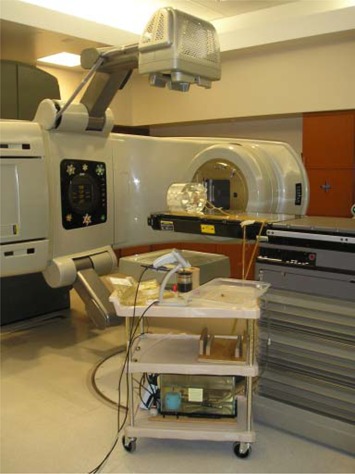
Phantom setup on treatment couch and in position for kilovoltage imaging. Heated water tank is present on cart.

Two DVS detectors were placed inside a commercially available acrylic pelvic phantom (Quasar, Modus Medical Devices, London, Ontario, Canada) (Fig. 2). Because the detectors' functionality and calibration is temperature‐dependent,^(6)^ ^,^ ^(15)^ ^,^ ^(16)^ heated water was circulated over them in a custom‐made insert to maintain their temperature at 37°±10°C (Fig. 3). The “center detector” was located in the center of the phantom, corresponding to the center of the prostate in a patient. The “edge detector” was located 2.5 cm posterior to the center detector, approximately corresponding to the posterior edge of the prostate in a patient. The detectors were located inside a larger cylindrical insert in the phantom, which made it possible to rotate the edge detector around the center detector.

Dose measurement using a DVS detector consists of telemetrically reading the MOSFET threshold voltage before (pre‐dose) and after (post‐dose) irradiation. The change in threshold voltage is then converted to dose based on the system calibration. In the current study, to exclude any imaging dose and to isolate for the treatment planned dose during each irradiation, imaging of the system was conducted before the pre‐dose reading was taken.

### B. Wedged‐field Plan

**Figure 4:**
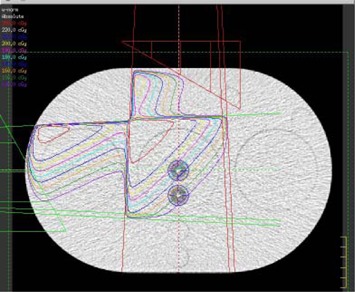
Wedged‐plan as described by the treatment planning system creating a uniform dose gradient across the two detectors. Isodose lines are in 5% dose steps. The detectors are the two white points (circled) near the center of the phantom.

The ability of the DVS detector to function as a combined dosimeter and fiducial marker, and the detector's ability to relate geometric misalignments with dosimetric errors, were first evaluated with a simple wedged‐field study using two 6 MV fields (Fig. 4). These fields created a relatively uniform and moderate dose gradient across the two detectors of nearly 1% of the dose per millimeter. In contrast to gradients encountered in clinical plans, this gradient was sufficiently steep that reasonable geometric shifts would correspond to a measurable dosimetric change but not so steep that phantom setup uncertainty could introduce substantial dosimetric errors. The planned doses were calculated using the Pinnacle treatment planning system (v.6.2b, Philips Medical Systems, Bothell, WA), with approximately 180 cGy delivered to the detectors. This planning system has been shown to accurately calculate dose distributions when properly commissioned.^(17)^

**Figure 5:**
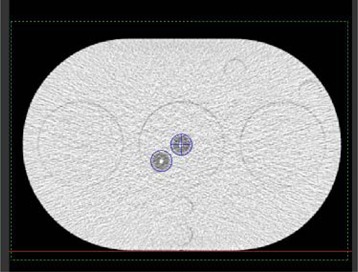
Alignment of the two detectors (circled) in the “rotated” configuration. Center detector remains in its original location; edge detector is rotated to approximately 45 degrees.

The wedged‐field plan was first developed for the phantom assuming the phantom was correctly aligned with its external markers and the dose delivered to the two detectors was calculated with the treatment planning system based on this “correct setup.” Three phantom setup errors were then considered: vertical misalignments of 0.5 cm posterior, 1.0 cm posterior, and a horizontal misalignment of 0.5 cm right. Shifts of these magnitudes are consistent with the observed interfractional motion of the prostate relative to either bony landmarks or skin marks.^(10)^ The expected dose was re‐calculated for each of these three incorrect setups. In addition to the three positional shifts, the dose delivered to the two detectors was also calculated assuming a correct alignment, but with the detectors rotated. This rotation simulated internal organ motion that may occur relative to the external markers. When rotated, the center detector was in the same location, but the edge detector was offset by approximately 45 degrees (Fig. 5).

**Figure 6:**
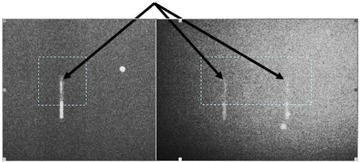
Anterior‐posterior (left) and lateral (right) portal images of the detectors as viewed in localization software. The dashed box and arrow in each image identifies the tip (active volume) of the detectors that was used for localization.

The five wedged‐field treatments (correct setup, 0.5‐cm vertical shift, 1.0‐cm vertical shift, 0.5‐cm lateral shift, and rotated detectors) were all delivered to the phantom using a Clinac 2100 accelerator (Varian Medical Systems, Palo Alto, CA) operating at 6 MV, and by moving the treatment couch as necessary. For each of the wedged‐field plans, the dose delivered was measured by each of the two implantable detectors. Additionally, for each treatment, an on‐board imaging (OBI) system was used to capture anterior‐posterior (AP) and lateral kilovoltage portal‐images of the phantom and the detectors. These images were imported into localization software (MarkerVision, Varian Medical Systems) (Fig. 6). The location of the tip of each detector was manually identified and compared to the planning computed tomography (CT) data set to determine the geometric deviation of the two detectors from their reference position (that is, from the correct alignment).

### C. Eight‐field Plan

**Figure 7:**
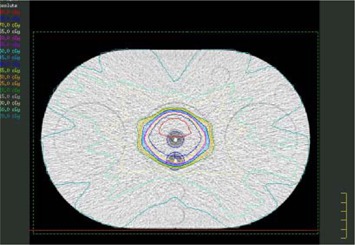
Eight‐field prostate treatment plan with homogeneous PTV and steep dose gradients. Isodose lines around the detectors are in 2.8% steps (5 cGy steps with a maximum dose of 180 cGy).

In addition to the wedged‐field plan, an 8‐field, conventional, 6 MV, prostate plan was developed for the phantom to test more clinically realistic conditions. The prostate plan delivered a uniform dose in the vicinity of the center detector. The posterior edge detector was also located in the uniform dose region, but at the very edge of the dose fall‐off gradient (Fig. 7). The detector locations relative to the high‐dose region were consistent with their placement during clinical trials.^(8)^ ^,^ ^(9)^

**Figure 8:**
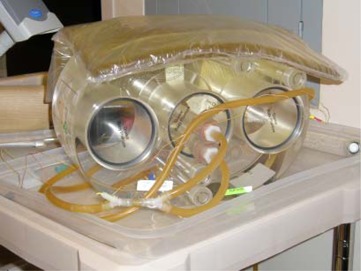
Phantom with 1 cm of bolus on top. Bolus was attached with tape and could be similarly taped to the bottom of the phantom.

Similar to with the wedged‐field plan, shifts were induced in the phantom relative to the external markers. These included shifts of 0.5 cm vertically (posterior), 0.5 cm laterally (right), and a rotation of the edge detector by approximately 45 degrees. A final shift that represented internal prostate motion was evaluated with two measurements. First, 1 cm of tissue‐equivalent bolus was placed on the bottom of the phantom. The phantom was then aligned correctly with its external markers. Second, without moving the treatment couch, the bolus was moved to the top of the phantom (Fig. 8). As a result, the external contour remained nearly constant, while the detectors moved 1 cm posterior within that external contour. For each treatment, the implantable detectors were used to measure the delivered doses and the OBI system was used to take AP and lateral images of the phantom to calculate the geometric displacement of the detectors from their reference position.

## III. RESULTS

### A. Wedged‐field Plan

The dose calculated by the treatment planning system and the dose measured by the implantable detectors for the five wedged‐field treatment plans are shown in Table 1. The measured dose showed good agreement with the calculated dose for both detectors for all alignments. The average deviation between measured and calculated dose was −1.4% for the center detector (standard deviation of 0.6%) and −1.3% for the edge detector (standard deviation of 1.2%). It should be emphasized that the percent difference shown in Table 1 is the difference between the measured dose and the dose predicted by the treatment planning software, not the difference introduced by shifting the detectors.

**Table 1:** Dosimetric comparison for wedged‐field plan with various misalignments

*Plan alignment/shifts*	*Center detector*			*Edge detector*		
	*Measured dose (cGy)*	*Calculated dose (cGy)*	*Difference*	*Measured dose (cGy)*	*Calculated dose (cGy)*	*Difference*
Correct alignment	175.4	178.3	−1.6%	148.3	150.1	−1.2%
Vertical shift, 0.5 cm	171.3	173.9	−1.5%	145.9	145.7	0.1%
Lateral shift, 0.5 cm	178.7	182.7	−2.2%	153.4	154.1	−0.5%
Vertical shift, 1.0 cm	167.3	169.2	−1.1%	137.2	141.1	−2.8%
Rotated detectors	181.2	182.2	−0.5%	174.4	178.3	−2.2%

The shifts in the detectors' positions introduced in each treatment plan are shown in Table 2, along with the detector shifts calculated by the localizing software. For most shifts of the phantom, a single overall shift was calculated for the two detectors because they did not move relative to each other. However, when the detectors were rotated, there was relative motion between them and the shift of each detector was calculated independently for this case. Good agreement was observed between the induced shifts and the calculated shifts. From both lateral and posterior shifts, it was possible to detect the induced shift within 0.11 cm at worst, and with a median detection of 0.09 cm. Unintended shifts never exceeded 0.12 cm (which was seen in the left‐right direction of the center detector during rotation).

**Table 2:** Geometric shifts as induced and as calculated by the localization software for the wedged‐field plan

*Plan alignment/shifts*	*Nominal induced shift (cm)*			*Calculated shift (cm)*		
	*LR*	*SI*	*AP*	*LR*	*SI*	*AP*
Correct alignment	0	00	−0.02	0.00	−0.04
Vertical shift, 0.5 cm	0	0 **0.5**	0.00	0.00	**0.41**
Lateral shift, 0.5 cm	**0.5**	00	**0.47**	0.00	−0.02
Vertical shift, 1.0 cm	0	0 **1.0**	−0.08	0.06	**0.89**
Rotation, Center detector	0	00	0.12	0.02	0.04
Rotation, Edge detector	−1.67	0	−0.90	−1.71	0.05	−0.80

LR=left‐right; SI=superior‐inferior; AP=anterior‐posterior.

The induced shifts and calculated shifts in the corresponding direction are shown in bold text.

### B. Eight‐field Plan

The dose calculated by the treatment planning system and the dose measured by the implantable detectors for the six 8‐field prostate treatment plans are shown in Table 3. As with the wedged‐field plans, the measured doses showed good agreement with the calculated doses for both detectors and all alignments except the “bolus on top” configuration. The average deviation between measured and calculated dose was −4.3% for the center detector (standard deviation of 1.2%) and −3.4% for the edge detector (standard deviation of 3.3%). The average percent difference and standard deviation were larger for the prostate plans than for the wedged‐field plans. A major source of this arose from the single deviant result that was obtained in the “bolus on top” configuration for the edge detector. If this data point is excluded, there is still a 2% average increase in the percent difference, but no statistically significant difference in the standard deviation between the results from the wedged‐field and prostate plans (as evaluated using an F‐test). The residual increase in the percent difference may be due to the more complex 8‐field plan, which would increase the uncertainty in both the delivered and calculated dose. Although the more complex plan was associated with a slightly larger dosimetric percent difference, studies have shown that the detector functions within manufacturer specified accuracy even for IMRT treatments.^(18)^

**Table 3:** Dosimetric comparison for 8‐field prostate treatment plan with various misalignments

*Plan alignment/shifts*	*Center detector*			*Edge detector*		
	*Measured dose (cGy)*	*Calculated dose (cGy)*	*Difference*	*Measured dose (cGy)*	*Calculated dose (cGy)*	*Difference*
Correct alignment	171.1	179.8	−4.8%	166.1	170.6	−2.6%
Vertical shift, 0.5 cm	172.7	179.3	−3.7%	140.8	145.6	−3.3%
Lateral shift, 0.5 cm	175.2	179.7	−2.5%	169.1	169.3	−0.1%
Rotated detectors	172.8	179.7	−3.8%	170.7	172.4	−1.0%
Bolus on bottom	169.9	178.4	−4.8%	159.9	166.0	−3.7%
Bolus on top	166.6	177	−5.9%	94.4	104.3	−9.5%

The detector shifts induced with each different setup alignment and those calculated by the localization software are shown in Table 4. As with the wedged‐field plans, a single shift is reported in Table 4 when the two detectors moved in synchrony while a separate shift was calculated for each detector for the rotated configuration. Good agreement was observed between the induced shifts and the calculated shifts, as it was possible to detect the induced shift within 0.16 cm at worst, and with a median detection of 0.09 cm. Unintentional shifts never exceeded 0.1 cm (in the superior‐inferior direction from the 0.5‐cm vertical shift). The localization system worked well to identify the detector locations regardless of whether the entire phantom was shifted (simulating setup errors) or the detectors moved relative to the external contour (simulating internal organ motion).

**Table 4:** Geometric shifts as induced and as calculated by the localization software for the 8‐field prostate treatment plan.

*Plan alignment/shifts*	*Nominal induced shift (cm)*			*Calculated shift (cm)*		
	*LR*	*SI*	*AP*	*LR*	*SI*	*AP*
Correct alignment	0	0	0	−0.05	0.04	−0.01
Vertical shift, 0.5 cm	0	0	**0.5**	0.00	0.10	**0.42**
Lateral shift, 0.5 cm	−0.5	00	−0.66	−0.01	−0.06
Rotation, Center detector	0	0	0	0.01	0.09	0.02
Rotation, Edge detector	−1.67	0	−0.90	−1.83	0.00	−0.88
Bolus on bottom	0	0	0	0.00	0.00	−0.02
Bolus on top	0	0	**1.0**	0.07	0.04	**0.91**

LR=left‐right; SI=superior‐inferior; AP=anterior‐posterior.

The induced shifts and calculated shifts in the corresponding direction are shown in bold text.

## IV. DISCUSSION

The implantable detector functioned well as both an internal dosimeter and an implantable fiducial marker.

Dosimetrically, the average difference between all measured and calculated doses was −2.7%. The systematic negative response is likely due to the calibration of the detectors as a single calibration is applied to a batch of detectors. As a result, individual detectors will have some small systematic bias. The 5.5% uncertainty cited by the manufacturer includes both the systematic error due to the calibration as well as random error in a given reading. In the current study, the worst dosimetric agreement was observed for the posterior edge detector during the 8‐field prostate study when bolus was placed on top of the phantom. This discrepancy between the measured and calculated dose likely arises from two sources. First, the nature of the shift was to move the posterior detector into the steep dose gradient region. In such a region, any small unintentional positioning errors (beyond the 1.0 cm shift) would correspond to substantial dosimetric errors. Second, the dose measured at this location (approximately 100 cGy), although consistent with the dose that would be encountered clinically for such organ motion during prostate radiation therapy, is outside of the calibration range of the dosimeters (150–250 cGy). Previously, at 100 cGy, the older generation implantable detectors were observed to under‐report the dose by 3.3%,^(19)^ which would partly account for the disagreement observed here.

The localization capabilities of this detector were also found to be good, agreeing at worst within 0.16 cm of the true value. The relatively small discrepancies that did occur were the result of uncertainty in the alignment of the BBs to the room lasers, uncertainty in the shift of the treatment couch (±0.05cm), and uncertainty in the manual localization of the detector tips in the localizing software images.

To avoid contaminating the treatment dose reading with dose from the imaging procedure, imaging was done prior to the pre‐dose reading of the MOSFET for each irradiation, as recommended.^(6)^ This is particularly appropriate as the DVS detectors have been shown to over‐respond to low energy (kV) photons as compared to MV energies.^(6)^ If imaging was conducted between the pre‐ and post‐dose reading, it may contribute to the dose read by the dosimeter. While the detectors will measure a notable dose from cone‐beam CT procedures, simple 2D kV imaging has been shown to produce a negligible response.^(6)^

Due to the design of the phantom, a limitation of the current study was that the dosimeters were always oriented parallel to the phantom axis (the detectors' long axes were perpendicular to the incident beams). This limitation has only a small impact in the current study because the manufacturer states that the detectors should be oriented in this fashion for clinical use. Provided that the detectors are placed within 30° of parallel to the body, dose perturbation resulting from beams intersecting the long axis of the detector is less than ±1.4%.^(6)^

There is clear utility in a single detector that offers both *in vivo* dosimetry and localization information. Beyond that, the detector may also relate, or at least offer insight into, the dosimetric impact of geometric errors such as misalignment, residual setup errors, or changes in the target shape. For the wedged‐field plan, based on the correct setup, the dose to the two detectors was known. If a geometric error was detected (either a setup error or internal motion), the dosimetric impact of the error was known and was clearly related to that geometric error because of the moderate and uniform dose gradient. However, such uniform dose gradients did not exist in the more clinically relevant 8‐field prostate plan and therefore no consistent connection between geometric shifts and dosimetric differences was seen. The center detector was in the middle of a uniform dose region and reported virtually identical doses for all geometric shifts, while the posterior edge detector sometimes showed a dosimetric difference when there was a geometric shift (such as with the vertical shift), but at other times showed none (such as with the lateral shift). The dosimeters used are point detectors and, as such, they are not necessarily able to capture the complex dose information of an altered dose‐volume histogram that would occur from any arbitrary geometric shift, such as patient setup error.

## V. CONCLUSIONS

The implantable detector functioned well as both a dosimeter and a fiducial marker and can therefore serve this dual purpose during clinical radiation therapy. The average dosimetric discrepancy between measurement and calculation was 2.7%, and the median and maximum alignment deviations were 0.09 cm and 0.16 cm respectively. As such, this dosimeter can serve as a useful clinical tool whereby the target location can be identified to facilitate patient repositioning, and the *in vivo* dose to the target can then be verified following irradiation.

## ACKNOWLEDGEMENTS

This work was supported by a grant from Varian Medical Systems. The authors would like to thank Gloria Beyer for her assistance and helpful discussions.
